# Assessing the psychological factors associated with vaccine hesitancy: psychometric properties of the psychological factors in healthcare behavior questionnaire (PF-HBQ)

**DOI:** 10.3389/fpubh.2026.1864623

**Published:** 2026-09-18

**Authors:** Rosalia De Biase, Laura Catalano, Francesco Panico, Isa Zappullo, Francesca D’Olimpio, Luigi Trojano, Laura Sagliano

**Affiliations:** Department of Psychology, University of Campania “Luigi Vanvitelli”, Caserta, Italy

**Keywords:** health behavior, healthcare decision-making, psychological factors, self-report questionnaire, vaccine hesitancy

## Abstract

**Introduction:**

Health-related decision-making is influenced by a complex interplay of cognitive, emotional, and personality-related factors. Psychological characteristics such as distress, personality traits, and maladaptive cognitive patterns play a central role in shaping how individuals perceive medical information, engage with healthcare systems, and adhere to health-related recommendations. Understanding these determinants is essential for clarifying individual differences in healthcare behaviors and for informing effective interventions in clinical and public health contexts. The present research introduces the Psychological Factors in Healthcare Behavior Questionnaire (PF-HBQ), a novel instrument designed to assess psychological dimensions relevant to healthcare-related decision-making. Overall, the PF-HBQ was developed to capture individual differences in psychological functioning that may influence attitudes and behaviors in medical contexts.

**Methods:**

The research was conducted through two studies. In Study 1, the psychometric properties of the questionnaire were evaluated on a large sample of individuals (N=472, 149 males, aged 18-86 years) by performing confirmatory and exploratory factor analysis, assessing test-retest reliability and convergent and divergent validity. In Study 2, differences in psychological factors between vaccine hesitant vs. non-hesitant individuals were explored in a further sample of participants (N=192, 47 males aged 18-71 years).

**Results:**

The results showed that the PF-HBQ questionnaire is a reliable and comprehensive tool for assessing 9 psychological factors (Suspiciousness, General Distress, Extraversion, Openness, Procrastination, Depressive Symptoms, Post-Traumatic Symptoms, Obsessive-Compulsive Traits, Emotional Stability) relevant to healthcare decision-making and shaping health-related attitudes and behaviors, including vaccination-related decision-making.

**Discussion:**

These findings may contribute to a deeper understanding of psychological influences on healthcare engagement and support the development of tailored public health strategies.

## 1 Introduction

Health-related behaviors play a central role in determining individual and population health outcomes. According to established classifications (1, 2), health-related behaviors can be categorized into health-promoting behaviors (e.g., physical activity, healthy diet, preventive screening, vaccination) and health-risk behaviors (e.g., substance use, non-adherence to treatments, sedentary lifestyle). This distinction provides a useful framework for a more comprehensive understanding of individual differences in health-related decision-making.

Recent theoretical developments such as the Personalized Health Determinants Model (3) highlight the dynamic interplay between psychological, behavioral, and contextual factors in shaping health outcomes. This model emphasizes the importance of considering individual-level psychological characteristics in interaction with environmental and social influences when examining healthcare behaviors.

Importantly, these individual differences may be partly explained by psychological factors, which can shape how individuals perceive, interpret, and respond to health-related information and situations (3, 4).

Psychological factors encompassing emotional states, cognitive processes, and personality traits play a central role in shaping multiple domains of individual functioning. These dimensions influence how individuals perceive, interpret, and respond to both internal experiences and external demands, by affecting behavior, decision-making, and interpersonal relationships. A large body of research within the biopsychosocial framework highlights that health outcomes are not only determined by biological mechanisms, but also by psychological and behavioral factors, including stress, beliefs, and coping strategies (5–7). Moreover, emotional and cognitive patterns, including anxiety, depression and maladaptive beliefs, further contribute to individuals’ functioning across contexts (8, 9).

Within the healthcare domain, these psychological factors have been shown to impact medical and health-related choices significantly, affecting treatment adherence, attitude toward medical recommendations and self-care behaviors (10, 11). Moreover, patients’ beliefs and emotional states can lead either to avoidance of necessary treatments or to maladaptive behaviors such as inconsistent or inappropriate medication use (12). More broadly, psychological factors also shape individuals’ attitudes toward healthcare systems, trust in medical professionals, and engagement in preventive behaviors, including vaccination and other health-promoting practices (13, 14).

In this context, vaccine hesitancy, defined as a refusal or delay in accepting vaccinations even when they are available (15), is one of the most significant health issues. This phenomenon has been documented since the first vaccine was available (16) but became so relevant in recent years because of global COVID-19 pandemic that the World Health Organization (WHO) defined it as a significant global health challenge (4, 17). Several studies showed that vaccine hesitancy is influenced by several factors, such as risk perception, safety concerns, perceived necessity, previous health experiences, sources of information, and ultimately influence of social networks (18–20). From a psychological perspective, a recent systematic review (4) showed that several psychological aspects are strictly related to vaccine hesitancy involving cognitive and emotional processes and personality traits (21–23). For instance, depressive symptomatology seems to be associated with higher vaccine hesitancy (24, 25). Moreover, stress has also been found to be positively related to vaccine reluctance (21, 25). The role of anxious symptomatology seems to be more controversial as higher levels of anxiety have reported to be associated with higher vaccine acceptance (21), whereas specific fears seem to be more associated with vaccine hesitancy (26). With respect to personality, some studies showed that stable dispositional traits, such as the ones described in the Big-Five framework (i.e., conscientiousness, neuroticism, extraversion, openness and agreeableness) and the ones outlined in the dark personality triad (i.e., psychoticism, narcissism and Machiavellianism) may influence hesitancy to vaccinate (4). Openness and agreeableness are negatively related to vaccine hesitancy, while extraversion seems to be positively associated with vaccine hesitancy (22). Moreover, a study from Giancola et al. (27) showed that the dark triad factors positively predict beliefs in conspiracy theories, that in turn, are directly associated with high vaccine hesitancy.

Based on the above-described evidence, the present work aims at identifying and assessing psychological factors that may be relevant within the healthcare domain. Specifically, we developed a novel questionnaire designed to evaluate a range of psychological dimensions that can influence individuals’ attitudes, expectations, and decision-making processes in medical contexts (Study 1). The selection of the psychological domains was based on the findings of recent systematic reviews (4, 9) highlighting the role of cognitive, emotional, and personality-related factors that influence medical decision-making. Specifically, the inventory incorporates scales to evaluate anxiety, depression, and stress symptoms, alongside obsessive-compulsive tendencies, narcissistic traits, paranoid ideation, post-traumatic stress symptoms, and broader personality traits. Furthermore, to examine the applicability of this instrument within a specific and highly relevant health context, Study 2 focused on vaccine hesitancy. In particular, the study aimed to investigate whether the psychological dimensions assessed by the questionnaire were associated with individual differences in attitudes toward vaccination, thereby providing an initial test of its utility in understanding vaccine-related decision-making.

These two studies allow to assess the relationship between all the psychological dimensions captured by the proposed scale and health-related decision making, with specific focus on attitude to vaccination, also testing whether specific personality profiles or distress levels can effectively predict an individual’s stance toward vaccinations.

### 1.1 Ethical aspects

All procedures adopted in the study were conducted according to the guidelines of the Declaration of Helsinki and approved by the ethical committee of the Department of Psychology, University of Campania “Luigi Vanvitelli” (39/2025).

## 2 Study 1

### 2.1 Materials and method

#### 2.1.1 Phase 1-conceptualization and item generation

The development and validation of the questionnaire followed established best practices for scale development as outlined by Boateng et al. (28). The process comprised sequential steps including item generation, content validity assessment, pre-testing (Phase 1), and large-scale psychometric evaluation (Phase 2).

Italian-language items were generated based on an extensive review of the literature on psychological determinants of health-related decision. In particular, the selection of the psychological domains was theoretically informed by recent systematic evidence highlighting the role of cognitive, emotional, and personality-related factors in shaping vaccine hesitancy (4). The initial item pool (*N* = 120) was designed to cover several psychological domains potentially relevant to health behavior, such as propension to vaccination, anxiety, depression, stress symptoms, obsessive-compulsive tendencies, narcissistic traits, paranoid ideation, post-traumatic stress symptoms, and personality traits. Items were formulated as clear, concise, and unambiguous self-report statements (28). Participants responded to each item on a 5-point Likert scale ranging from 1 (“completely disagree”) to 5 (“completely agree”).

The final Italian version of the scale, including administration and scoring modality, and its English translation are reported in the Supplementary materials.

#### 2.1.2 Content validity

Content validity was assessed through an anonymous expert evaluation procedure (28). Specifically, the preliminary pool of items was submitted to a panel of 5 experts in psychology and health-related research. The panel included clinical psychologists and university professors with experience in psychological assessment and health psychology. Experts were asked to rate the extent to which each item adequately represented the intended psychological construct using a four-point relevance scale (1 = not at all, 2 = slightly, 3 = moderately, 4 = fully). In addition, a free-text comment section was provided for each item, allowing experts to report qualitative feedback, suggest rewording, or highlight conceptual or linguistic issues. The evaluation was conducted online using PsyToolkit (29), ensuring anonymity of responses and independent judgments.

Content validity was quantified using the Item-Level Content Validity Index (I-CVI; (28)), calculated as the proportion of experts rating the item as moderately or highly relevant to each psychological construct (scores of 3 or 4). Items not meeting such conventional adequacy thresholds were revised or removed. Quantitative ratings and qualitative feedback were jointly considered to guide item refinement. Following experts’ evaluation, one item assessing narcissistic traits was excluded due to inadequate content validity.

#### 2.1.3 Pre-testing

During this phase, a novel group of experts (*N* = 5) was asked to complete the draft questionnaire and to evaluate whether each item was appropriate and comprehensible with respect to the intended psychological construct, providing further suggestions for modifications in wording, syntax, item formulation, or response scale. This pre-testing procedure allowed the identification of potential misunderstandings, ambiguous formulations, and suboptimal response options. The finalized version of the questionnaire was derived from the combined results of the expert evaluation and the pre-testing phase.

#### 2.1.4 Phase 2–survey administration and psychometric assessment

##### 2.1.4.1 Procedure and experimental design

The final version of the questionnaire, termed *Psychological Factors in Healthcare Behavior Questionnaire (PF-HBQ),* was administered using a mixed-mode approach, combining an online self-administration implemented via PsyToolkit (29) and a paper-and-pencil completion format. The assignment to either completion mode was based on participants’ preferences and practical accessibility. The use of mixed-mode data collection has been recommended in survey research as a strategy to minimize measurement error, reduce data entry inaccuracies, and increase sample accessibility and coverage, while mitigating potential biases associated with single-mode administration (30).

All participants received information about the study aims and provided their informed consent prior to completing the questionnaire, either electronically or in written form.

First, participants were asked to fill an initial section, specifically developed to collect demographic characteristics (age, gender, educational level, occupational status, economic status, marital status and residence) and health-related information (general medical conditions, high-risk medical conditions, vaccination uptake, adverse reactions). This initial section is reported in Supplementary materials. Then, they compiled the *PF-HBQ* together with a set of validated questionnaires that measures several psychological dimensions for the assessment of construct validity (described below).

##### 2.1.4.2 Participants

The initial sample consisted of 472 individuals (140 males) aged between 18 and 86 years (M = 35.18; SD = 13.55), participating voluntarily and anonymously to the study. Recruitment followed a snowball sampling method through investigators’ acquaintances or social media, and participants were university students, unemployed, retired or workers of the public or private sectors. The sample size was considered adequate for the planned psychometric analyses, including exploratory and confirmatory factor analyses (28, 31). Eligibility criteria included being at least 18 years old.

For factor analytic purposes, the final dataset was split into two independent subsamples. Even-numbered cases were allocated to the Exploratory Factor Analysis (EFA) whereas odd-numbered cases were allocated to the Confirmatory Factor Analysis (CFA).

#### 2.1.5 Data analysis

Before performing statistical analyses, a quality check of the responses was performed. Participants were excluded if they: (i) did not answer to all items of the questionnaire (more than 1 unanswered item), (ii) spent excessively long time to complete it (more than 1.5 h), (iii) failed to correctly pass all the attention-check items embedded in the questionnaire (i.e., “Please respond to this item with “Completely agree”).

Exploratory Factor Analysis (EFA) and internal consistency analyses (Cronbach’s alpha) were conducted using Jamovi (version 2.6.44), while Confirmatory Factor Analysis (CFA) was performed using RStudio (version 2024.12.0. + 467). All remaining statistical analyses were carried out using IBM SPSS Statistics (version 21).

##### 2.1.5.1 Exploratory factor analysis

Exploratory Factor Analysis (EFA) was conducted to examine the latent structure of the *PF-HBQ*. Prior to factor extraction, multivariate normality was assessed using Mardia’s test. The results indicated a significant deviation from multivariate normality (multivariate skewness: *p* < 0.001; multivariate kurtosis: *p* < 0.001). Consequently, factor extraction was performed using the minimum residual method, a least-squares-based factor extraction approach that minimizes residual correlations (32, 33). Sampling adequacy was evaluated through the Kaiser–Meyer–Olkin (KMO) index and Bartlett’s test of sphericity. KMO values above 0.60 were considered acceptable, whereas values above 0.80 indicated good sampling adequacy (34). The number of factors to retain was determined by combining parallel analysis, inspection of the scree plot, and theoretical interpretability of the resulting solution (35, 36). Because the psychological constructs assessed were conceptually related and expected to show non-zero intercorrelations, an oblique rotation (Oblimin rotation) was applied (32). Items with factor loadings below 0.30, substantial cross-loadings, or low communalities were considered for removal (37). To further assess factor retention, Velicer’s Minimum Average Partial (MAP) test was conducted, together with the Very Simple Structure (VSS) criterion. Decisions regarding item retention were guided by both statistical criteria and theoretical coherence to ensure a parsimonious and interpretable factor structure.

##### 2.1.5.2 Confirmatory factor analysis

The factor structure identified in the exploratory phase was tested using CFA. Given the ordinal nature of the Likert-type response scales, the model was estimated using the Weighted Least Squares Mean and Variance adjusted (WLSMV) estimator, which is recommended for categorical or ordinal indicators and provides robust parameter estimates and standard errors (38). Model fit was assessed using several indices: the chi-square goodness-of-fit statistic, the Comparative Fit Index (CFI), the Tucker-Lewis Index (TLI), the Root Mean Square Error of Approximation (RMSEA), and the Standardized Root Mean Square Residual (SRMR). Based on the criteria proposed by Hu and Bentler (39), the model fit indices were considered good if χ^2^/df ≤ 2, RMSEA ≤ 0.08, SRMR ≤ 0.08, and CFI and TLI ≥ 0.95.

##### 2.1.5.3 Internal consistency

Internal consistency of the retained factors was assessed using Cronbach’s alpha and McDonald’s omega coefficients. Reliability values of 0.70 or higher were considered acceptable (40).

##### 2.1.5.4 Test–retest reliability

To assess temporal stability, a subsample of available participants completed the *PF-HBQ* a second time after the specified interval for test–retest reliability analyses. Test–retest reliability was estimated using the intraclass correlation coefficient (ICC), a statistic that quantifies the degree to which repeated measurements obtained from the same individuals are comparable relative to between-subject variability (41). Interpretation of ICC values followed established guidelines (42): coefficients below 0.50 were considered indicative of poor reliability, values between 0.50 and 0.75 reflected moderate stability, values between 0.75 and 0.90 indicated good reliability, and coefficients exceeding 0.90 were interpreted as evidence of excellent temporal stability.

##### 2.1.5.5 Construct validity

Convergent validity was assessed through correlations with validated instruments capturing psychological dimensions of interest. Specifically, emotional distress was measured using the Depression Anxiety Stress Scales–21 (DASS-21; (43)). The DASS-21 consists of 21 items, each rated on a 4-point Likert scale. The general distress score ranges from 0 to 63, with higher scores indicating higher level of distress. Obsessive-compulsive symptoms were assessed with the Obsessive-Compulsive Inventory–Revised (OCI-R; (44)). It consists of 18 items, each rated from 0 to 4 on a 5-point Likert scale. The total score ranges from 0 to 72; higher scores indicate greater levels of obsessive-compulsive traits. The trauma-related symptomatology was evaluated using the National Stressful Events Survey PTSD Short Scale (NSESSS; (45)). It consists of 9 items, each answered on a 5-point Likert scale ranging from 0 to 4. The mean score ranges from 0 to 4; higher scores indicate higher severity of post-traumatic symptoms. Persecutory ideation was measured through the Persecutory Ideation Questionnaire (PIQ; (46, 47)). It consists of 10 items, each rated from 0 to 4 on a 5-point Likert scale. The total score ranges from 0 to 40. Higher scores indicate higher levels of persecutive ideation. Finally, personality traits were assessed using the Big Five Inventory–10 (BFI-10; (48)) that measures 5 subscales: *Agreeableness, Conscientiousness, Emotional Stability, Extroversion* and *Openness*. It consists of 10 items, each rated from 1 to 5 on a 5-point Likert scale. Each subscale score ranges from 2 to 10 with higher level indicating higher personality traits. Moderate and statistically significant correlations in theoretically expected directions were interpreted as evidence supporting convergent validity.

Divergent validity was assessed through the Interpersonal Reactivity Index that measures 4 subscales: *Fantasy, Perspective Taking, Empathic Concern and Personal Distress*. It consists of 28 items, each rated from 0 to 4 on a 5-point Likert scale. Each subscale score ranges 0 to 28 with higher scores indicating higher levels of empathic dimensions (IRI; (49)).

##### 2.1.5.6 Differential item functioning analyses on demographic characteristics and comparison between paper-version and online-survey respondents

To assess whether the modality of data collection was associated with demographic aspects, thereby ruling out potential methodological biases associated with the administration format, three Chi-squared tests were computed to compare paper-version and Online-survey respondents in demographic characteristics (age, gender and educational level). The level of significance was set at *p* < 0.05. To examine whether individual PF-HBQ items functioned equivalently across participant characteristics and administration formats, differential item functioning (DIF) analyses for ordinal data were conducted separately for each subscale. Cumulative ordinal logistic models were estimated using the standardized subscale score as the matching criterion (50, 51). Gender, age, educational level, and administration format were entered as binary grouping variables. Specifically, gender was coded as *Male* vs. *Female* (two participants who preferred not to report their gender were excluded from these analyses), age as *Young* (participants aged <40 years) vs. *Adult* (participants aged ≥40 years), educational level as *non-Tertiary* (years of schooling ≤13) vs. *Tertiary* (years of schooling >13), and administration format as *Online* vs. *Paper-based* completion. Omnibus, uniform and non-uniform DIF were examined through likelihood-ratio tests, and Benjamini–Hochberg correction was applied to control for the false discovery rate within each subscale (50, 51).

## 3 Results

### 3.1 Descriptive data on the sample

From the initial sample of 503 participants, 16 participants were excluded from the analyses due to excessively long response durations, likely suggesting inattentive or invalid responding. Additionally, 10 participants were excluded because they failed the instructed responses (attention-check) item embedded in the questionnaire. Finally, 3 participants were excluded due to partial completion of the questionnaires.

After data screening procedures, the final sample comprised 472 participants aged between 18 and 86 years (M = 35.18 years, SD = 13.75). The sample included 330 females (M = 35.25 years, SD = 13.75), 140 males (M = 34.88 years, SD = 13.55), and 2 individuals who preferred not to report their gender (M = 46.5 years, SD = 11.19). Twenty-six participants had educational levels from elementary to middle school, 194 participants had achieved a high school diploma, 235 had achieved a bachelor’s or master’s degree and 17 had a PhD or an equivalent degree. Detailed description of the sample is described in Table 1.

**Table 1:** Demographic characteristics of the sample in Study 1.

Educational level	*N*	Gender	Mean age	Religion				Economic status				Residence		
				Atheist/Agnostic	Cristian	Hinduist	Baha’i	<20.000€	20.000–40.000€	40.000–60.000€	>60.000€	Countryside	Town	City
Elementary School	2	2 females	49	50%	50%	—	—	50%	50%	—	—	—	100%	—
Middle School	24	15 females; 9 males	48.45	37.5%	62.5%	—	—	87.5%	12.5%	—	—	13.04%	78.26%	8.7%
High School	194	130 females; 63 males; 1 other	35.19	71.65%	27.83%	0.52%	—	46.91%	41.24%	9.28%	2.57%	8.25%	72.16%	19.59%
Bachelor Degree	92	73 females; 18 males; 1 other	28.82	73.92%	26.08%	—	—	51.07%	38.03%	8.7%	2.2%	6.52%	70.65%	22.83%
Master Degree	143	99 females; 44 males	36.57	79.03%	20.27%	—	0.7%	37.06%	48.25%	8.3%	6.3%	6.99%	63.64%	29.37%
PhD or equivalent	17	11 females; 6 males	37.76	76.48%	23.52%	—	—	29.41%	41.18%	11.76%	17.65%	—	29.42%	70.58

The even-numbered subset for EFA included 236 participants (169 females, 65 males, and 2 who preferred not to declare their sex; M = 35.11 years, SD = 13.64). The odd-numbered cases for CFA included 236 participants (161 females; M = 35.27 years, SD = 13.89). The subsample available to assess temporal stability was composed of 142 participants (87 females; M = 37.38 years, SD = 13.72).

### 3.2 Exploratory factor analysis

EFA was conducted on the 119 original items. Inspection of the factor loadings revealed that several items (N = 65) showed factor loadings below 0.30 or substantial cross-loadings, which limited interpretability (36, 37). These items were removed and a second EFA was then conducted on the remaining 54 items. Sampling adequacy indicated that the data were suitable for factor analysis (χ^2^ (1431) = 5,261, *p* < 0.001; KMO = 0.82). This analysis yielded a more parsimonious and interpretable 9-factor model that explained 44.49% of the common variance. Although parallel analysis supported the nine-factor solution, additional factor retention methods provided partially divergent recommendations. Specifically, Velicer’s MAP test suggested a six-factor solution, whereas VSS indicated a three-factor solution. Thus, the retention criteria did not converge on a single factorial structure. To evaluate these alternative models, additional EFAs were examined using the minimum residual method and oblique rotation (Oblimin). The six-factor solution accounted for 38.02% of the total variance, whereas the three-factor solution explained only 28%. Beyond the lower amount of total variance explained, both the three-factor solution and the six-factor solution resulted in a simple structure that was not very satisfactory, including cross-loadings and several items without a salient loading of at least 0.30. In detail, the six-factor solution would imply the loss of additional eight items, with the resulting factors comprising 4 to 12 items. The three-factor solution would lead to exclusion of further 10 items, with the resulting factors comprising 5 to 32 items. More importantly, forcing inclusion of items into 6 or 3 factors would combine conceptually distinct domains within the same factors, substantially reducing interpretability and distinctiveness of the resulting dimensions. Therefore, considering the lack of convergence among the statistical retention criteria, the clarity of factor loading and the theoretical coherence of the competing alternatives, the nine-factor model was deemed as the most robust, meaningful, and comprehensive representation of the construct.

### 3.3 Confirmatory factor analysis

The initial CFA conducted on the 9-factor solution derived from the EFA showed adequate model fit (RMSEA = 0.077; CFI = 0.943; TLI = 0.940; SRMR = 0.097). One item (Item 118) had weak factor loadings (0.299) so it was removed to improve the interpretability and parsimony of the measurement model (52). The final model consisted of 53 items loading on 9 factors (identified as *Suspiciousness, General Distress, Extraversion, Openness, Procrastination, Depressive Symptoms, Post-Traumatic Symptoms, Obsessive-Compulsive Traits, Emotional Stability*), showing good and acceptable fit indices (RMSEA = 0.075, 90% CI [0.071, 0.078]; CFI = 0.949; TLI = 0.945; SRMR = 0.0984; χ^2^(1289) = 2153.40, *p* < 0.001). Standardized factor loadings ranged from 0.37 to 0.92 (see Figure 1).

**Figure 1:**
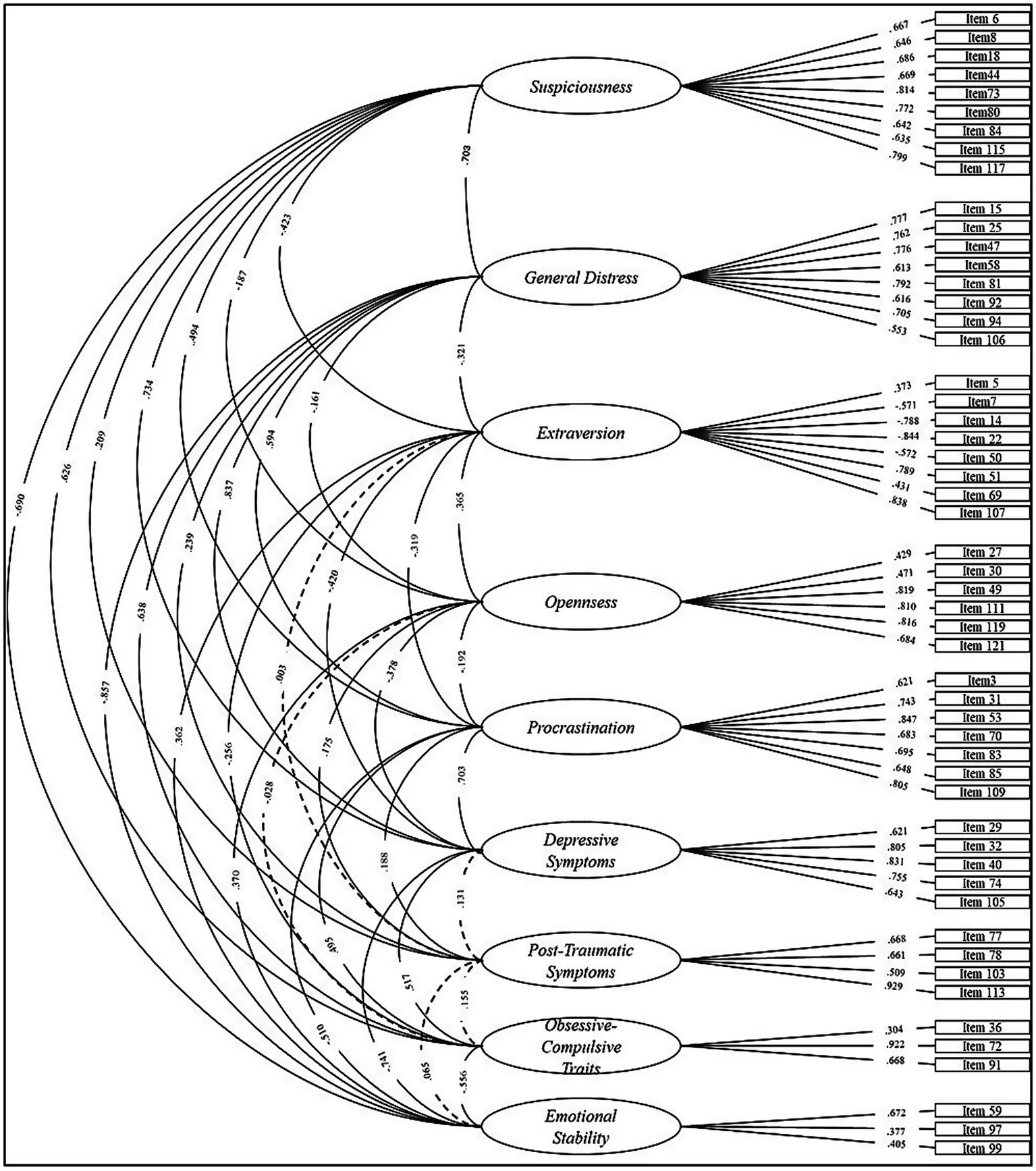
Factor loadings of 53-items (continuous lines represent significant correlations; dotted lines represent non-significant correlation).

### 3.4 Internal consistency and test–retest reliability

The internal consistency of the questionnaire was assessed using both Cronbach’s alpha (α) and McDonald’s omega (ω) coefficients for each subscale. Overall, the results indicated from acceptable to good reliability for most dimensions. The alpha coefficient ranged from 0.52 to 0.85 and the omega coefficient ranged from 0.52 to 0.86 for the 9 subscales and the total score. The test–retest reliability analysis showed a moderate and good reliability over time with ICCs ranging from 0.63 to 0.88. All indices are reported in Table 2.

**Table 2:** Mean (M), standard deviation (SD), cronbach alpha, omega coefficient at first administration (ω1) and retest (ω2), and intraclass correlation coefficient (ICC) with confidence intervals (CI) for the total score and the 9 subscales.

Scales	M	SD	𝛼	𝜔1	𝜔2	ICC	95% CI
Suspiciousness	2.17	±0.77	0.84	0.85	0.86	0.87	0.82–0.90
General distress	3.02	±0.94	0.85	0.86	0.87	0.85	0.79–0.89
Extraversion	3.30	±0.87	0.82	0.82	0.81	0.88	0.84–0.91
Openness	3.27	±0.77	0.74	0.76	0.72	0.78	0.71–0.84
Procrastination	2.56	±0.86	0.81	0.82	0.81	0.81	0.84–0.91
Depressive symptoms	2.10	±0.88	0.79	0.79	0.85	0.82	0.76–0.87
Post-traumatic symptoms	1.93	±0.97	0.66	0.67	0.67	0.77	0.69–0.83
Obsessive-compulsive traits	2.28	±1	0.57	0.64	0.74	0.81	0.75–0.86
Emotional stability	3.50	±0.75	0.52	0.52	0.47	0.63	0.51–0.72

### 3.5 Convergent validity

As reported in Table 3, correlational analyses largely supported the convergent validity of the *PF-HBQ*. Only the strong and moderate associations of primary theoretical interest (*r* > ±0.40) are described below, while the complete correlation matrix is reported in Table 3.

**Table 3:** Correlations between the 9-factors and psychological measures.

Scales	Big-five conscientiousness	Big-five emotional stability	Big-five extraversion	Big-five openness	OCI-R	NSESSS	DASS-21	PIQ
Suspiciousness	−0.25^**^	−0.42^**^	−0.23^**^	0.01	0.46^**^	0.46^**^	0.51^**^	0.71^**^
General Distress	−0.26^**^	−0.64^**^	−0.15^**^	0.14	0.52^**^	0.60^**^	0.67^**^	0.32^**^
Extraversion	0.20^**^	0.20^**^	0.78^**^	0.17	−0.22^**^	0.00	−0.16^**^	−0.09^*^
Openness	0.14^**^	0.15^**^	0.27^**^	0.51^**^	0.02	0.02	−0.12^*^	0.02
Procrastination	−0.66^**^	−0.30^**^	−0.12^*^	0.21^*^	0.32^**^	0.34^**^	0.46^**^	0.25^**^
Depressive Symptoms	−0.41^**^	−0.42^**^	−0.22^**^	0.06	0.37^**^	0.44^**^	0.61^**^	0.37^**^
Post-Traumatic Symptoms	−0.02	0.04	0.09^*^	0.19^*^	0.12^**^	0.24^**^	0.13^**^	0.18^**^
Obsessive-Compulsive Traits	−0.07	−0.30^**^	−0.09	0.19^*^	0.64^**^	0.34^**^	0.33^**^	0.26^**^
Emotional Stability	0.24^**^	0.56^**^	0.10^*^	−0.06	−0.30^**^	−0.25^**^	−0.34^**^	−0.29^**^

*p < 0.05; **p < 0.01; OCI-R, obsessive compulsive inventory revised; NSESSS, national stressful events survey PTSD short scale; DASS-21, depression anxiety stress scale; PIQ, persecutory ideation questionnaire.

The *Suspiciousness* factor was strongly and positively correlated with persecutory ideation (PIQ; *r = 0.71; p < 0.01*), as well as with OCI-R, NSESSS, and negatively with Emotional Stability. The *General Distress* factor showed a strong positive correlation with DASS-21 (*r* = 0.67; *p* < 0.01) and was also positively associated with NSESSS and OCI-R, while being negatively correlated with Emotional Stability (*r* = −0.64; *p* < 0.01). The *Extraversion* factor was strongly and positively associated with the BFI-10 Extraversion dimension (*r = 0.78; p < 0.01*). The *Openness* factor showed a strong positive correlation with the BFI-10 Openness dimension (*r* = 0.51; *p* < 0.01). The *Procrastination* factor was strongly negatively correlated with Conscientiousness (*r* = −0.66; *p* < 0.01) and positively correlated with DASS-21. The *Depressive Symptoms* factor showed a strong positive correlation with DASS-21 (*r* = 0.61; *p* < 0.01) and was also negatively associated with Conscientiousness and Emotional Stability and positively with NSESSS. The *Post-Traumatic Symptoms* factor was positively correlated with NSESSS (*r* = 0.24; *p* < 0.01). The *Obsessive-Compulsive Traits* factor showed a strong positive association with OCI-R (*r* = 0.64; *p* < 0.01). Finally, *Emotional Stability* was positively correlated with the BFI-10 Emotional Stability dimension (*r* = 0.56; *p* < 0.01).

### 3.6 Divergent validity

To examine divergent validity, correlations between the *PF-HBQ* factors and empathy dimensions assessed by the *Interpersonal Reactivity Index* (*IRI; Fantasy, Perspective Taking, Empathic Concern, and Personal Distress*) were computed (see Table 4). The factors *Extraversion, Openness, Procrastination, Depressive Symptoms, and Post-Traumatic Symptoms* were not significantly associated with *Empathic Concern* (*p* > 0.05). However, weak correlations were observed with *General Distress, Depressive Symptoms, Procrastination, Suspiciousness* and *Personal Distress*, as well as between *Emotional Stability* and both *Personal Distress* and *Perspective Taking*.

**Table 4:** Correlations between the 9-factors and interpersonal reactivity index subscale.

Scales	IRI-F	IRI-PT	IRI-EC	IRI- PD
Suspiciousness	0.19^**^	−0.17^**^	−0.12^**^	0.34^**^
General distress	0.35^**^	0.11^*^	0.15^**^	0.50^**^
Extraversion	−0.04	0.05	0.16^**^	−0.20^**^
Openness	0.21^**^	0.10^*^	0.07	−0.19^**^
Procrastination	0.29^**^	0.03	−0.08	0.29^**^
Depressive symptoms	0.15^**^	−0.03	−0.05	0.40^**^
Post-traumatic symptoms	0.06	0.04	0.02	−0.06
Obsessive-compulsive traits	0.21^**^	−0.08	0.04	0.26^**^
Emotional stability	−0.10^*^	0.24^**^	0.12^*^	−0.46^**^

*p < 0.05; **p < 0.01; IRI-F, interpersonal reactivity index-fantasy subscale; IRI-PT, interpersonal reactivity index perspective-taking subscale; IRI-EC, interpersonal reactivity index-empathic concern subscale; IRI- PD, Interpersonal reactivity index-personal distress subscale.

### 3.7 Demographic differences between paper-version and online-survey respondents

Chi-squared test computed on age group showed significant results (χ^2^ = 23.1, *p* < 0.001, Cramer’s V = 0.22; Paper-version: Young *n* = 66, Adult *n* = 61; Online-survey: Young *n* = 259, Adult *n* = 86). Chi-squared test computed on education group showed significant results (χ^2^ = 98.9, *p* < 0.001, Cramer’s V = 0.45; Paper-version: Tertiary *n* = 107, non-Tertiary *n* = 20; Online-survey: Tertiary *n* = 113, non-Tertiary *n* = 232). Chi-squared test computed on gender showed no significant results (χ^2^ = 0.775, *p* = 0.679, Cramer’s V = 0.041; Paper-version: Male *n* = 37, Female *n* = 90, Other *n* = 0; Online-survey: Male *n* = 103, Female *n* = 240, Other *n* = 2).

### 3.8 Differential item functioning analyses

Item functioning was largely comparable across groups, although some item-specific differences emerged. After Benjamini–Hochberg correction, gender-related DIF was detected for six items: Item 47 within General Distress factor; Items 7, 50, and 69 within Extraversion factor; and Items 78 and 103 within Post-Traumatic Symptoms. Age-related DIF was more widespread and involved 13 items across Suspiciousness (Items 73 and 115), Extraversion (Items 5, 7, and 51), Openness (Items 27 and 121), Procrastination (Items 85 and 109), Obsessive-Compulsive Traits (Items 36 and 72), and Emotional Stability (Items 59 and 99) factors. Educational-level DIF was observed for 2 items: Items 31 and 83 within Procrastination factor. Finally, administration-format DIF emerged for 3 items: Item 30 within Openness, Item 53 within Procrastination, and Item 59 within Emotional Stability factor. No significant DIF was identified for the remaining items after correction for multiple comparisons. Detailed likelihood-ratio statistics, adjusted *p*-values, and the classification of significant effects as uniform or non-uniform are reported in the Supplementary Tables S1–S4.

### 3.9 Summary of study 1

Results from Study 1 showed that the *PF-HBQ* demonstrated overall good psychometric properties. In particular, the analyses revealed a structure in line with the theoretically hypothesized dimensions and satisfactory levels of internal consistency. Given that the *PF-HBQ* assesses general psychological aspects rather than constructs that are specifically referred to the medical context, a further investigation of the relationship between this scale and vaccination attitudes was carried out. Based on these considerations, Study 2 was specifically designed to explore these aspects.

## 4 Study 2

### 4.1 Materials and method

Study 2 assessed differences in psychological factors as measured by the *PF-HBQ* between hesitant and non-hesitant participants. Data collection was carried out with *PsyToolkit* online platform (29), exclusively through an online self-administration format (no paper-based version). As in the Study 1, recruitment followed a snowball sampling method through investigators’ acquaintances or social media, and participants were university students, unemployed, retired or workers of the public or private sectors. Eligibility criteria included being at least 18 years old.

#### 4.1.1 Procedure

Prior to participation, participants provided their informed consent. Then participants were asked to report information about demographic characteristics and medical conditions (see Supplementary materials). Data were collected using two instruments: the *PF-HBQ* and the Italian version of the *Vaccination Attitudes Examination scale* (VAX-I; (53)). The VAX-I consists of 12 items each rated on a 6-point Likert-scale (1 = strongly disagree to 6 = strongly agree) that measure attitude towards vaccination (e.g., mistrust of vaccine benefit, worries about unforeseen future effects, concerns about commercial profiteering and preference for natural immunity). The average completion time was approximately 20 min.

#### 4.1.2 Participants

The sample consisted of 192 participants aged between 18 and 71 years (M = 34.83 years, SD = 12.31) not involved in Study 1. The sample included 145 females (M = 33.42 years, SD = 11.71), 47 males (M = 39.17 years, SD = 13.19). Nine participants had middle school license, 74 participants had a high school diploma, 104 had achieved a bachelor’s or master’s degree and 5 had a PhD or an equivalent degree. Based on the median score of the VAX-I (median = 2.00), the sample was divided into two groups: a lower hesitancy group (*N* = 101) and a higher hesitancy group (N = 91). Participants were also classified into two groups based on their responses to general vaccine-related items of the initial interview. The VAX group included individuals who had received both mandatory and recommended vaccinations, whereas the UNVAX group included individuals who had received only some, or none, of the recommended vaccinations, and in some cases had also not received all the mandatory vaccinations.

#### 4.1.3 Statistical analyses

All statistical analyses were performed using SPSS software v.25. A MANOVA was conducted to examine differences between the two groups based on VAX-I scores across the following dependent variables as measured by the newly developed *PF-HBQ*: *Suspiciousness, General Distress, Extraversion, Openness, Procrastination, Depressive Symptoms, Post-Traumatic Symptoms, Obsessive-Compulsive Traits,* and *Emotional Stability*. Since the VAX (*n* = 139) and UNVAX (*n* = 53) groups were numerically unbalanced, binary logistic regressions were employed as the most appropriate analytic approach (54, 55). The dependent variable was vaccination-group (0 = VAX, 1 = UNVAX), and the predictors included *Suspiciousness, General Distress, Extraversion, Openness, Procrastination, Depressive Symptoms, Post-Traumatic Symptoms, Obsessive-Compulsive Traits, and Emotional Stability*. All predictors were entered simultaneously using the enter method. Similarly, a further binary logistic regression was computed with vaccination-group as dependent variable and VAX-I score as predictor. The level of statistical significance was set at *p* < 0.05.

## 5 Results

The MANOVA conducted to examine differences between low and high vaccine hesitancy groups on psychological factors revealed that individuals with high vaccine hesitancy reported higher levels of *Suspiciousness, Extraversion*, and *Obsessive-Compulsive Traits* compared to individuals with lower hesitancy levels. In contrast, individuals with lower vaccine hesitancy showed higher levels of *Emotional Stability*. No significant differences emerged for the remaining variables. Full statistics are reported in Table 5.

**Table 5:** Statistics from MANOVA conducted to examine differences between low and high vaccine hesitancy groups.

	Pillai’s trace	*F*	*p*-value	η_p_^2^
Multivariate test				
Group VAX-I	0.147	3.485	<0.001	0.147

	Low-hesitance group (*N* = 101)	High-hesitance group (*N* = 91)	*F*	*p*-value	η_p_^2^
Between-subject effects					
Suspiciousness	1.74 ± 0.66	2.00 ± 0.76	6.647	0.011	0.034
General distress	2.60 ± 0.98	2.69 ± 1.04	0.46	0.499	0.002
Extraversion	3.24 ± 0.66	3.41 ± 0.51	3.987	0.047	0.021
Openness	2.99 ± 0.69	3.07 ± 0.75	0.582	0.446	0.003
Procrastination	2.55 ± 0.82	2.46 ± 0.81	0.593	0.442	0.003
Depressive symptoms	1.90 ± 0.85	2.02 ± 0.86	0.796	0.373	0.004
Post-traumatic symptoms	1.69 ± 0.88	1.81 ± 0.93	0.832	0.363	0.004
Obsessive-compulsive traits	1.83 ± 0.78	2.11 ± 0.91	5,141	0.024	0.026
Emotional stability	3.46 ± 0.74	3.21 ± 0.83	4,784	0.03	0.025

The logistic regression models demonstrated that the *PF-HBQ* psychological variables [χ^2^(9) = 9.15, *p* = 0.424; Nagelkerke *R*^2^ = 0.067; Cox & Snell *R*^2^ = 0.047] and VAX-I score [χ^2^(1) = 3.246, *p* = 0.072; Nagelkerke *R*^2^ = 0.024; Cox & Snell *R*^2^ = 0.017] were not significant predictors of the group membership; therefore, these scales could not reliably distinguish between vaccinated and unvaccinated participants.

## 6 Discussion

Psychological factors encompassing emotional states, cognitive processes, and personality traits have been widely recognized as central determinants of health-related decision-making (9). Within health-related issues, vaccine hesitancy represents a major global health concern (56), that is shaped by several psychological dimensions (4, 21–23, 57). The present study contributes to this issue by developing and validating a novel Italian questionnaire, the *PF-HBQ*, designed to assess psychological factors associated with health-related decision-making.

Overall, the findings show that the *PF-HBQ* has robust psychometric properties. Exploratory and confirmatory factor analyses converged on a coherent 9-factor structure, characterized by acceptable model fit, adequate standardized loadings, and satisfactory internal consistency across dimensions. Furthermore, test–retest analyses indicated moderate to good temporal stability, suggesting that the identified factors reflect relatively stable psychological characteristics rather than transient fluctuations.

Convergent validity analyses provided strong support for the construct validity of the instrument. Several dimensions showed robust and theoretically coherent associations with external measures. At the same time, the correlation matrix revealed a pattern of moderate cross-associations among several dimensions, particularly involving distress-related, obsessive-compulsive, and trauma-related variables. Rather than indicating a lack of specificity, this pattern is consistent with dimensional models of psychopathology, which posit that internalizing symptoms share a common underlying vulnerability (58–61). The relationship between procrastination and distress-related measures can be interpreted within frameworks linking avoidance tendencies and emotional dysregulation (62, 63). Overall, the observed correlational structure supports the convergent validity of the instrument while reflecting the inherently interconnected nature of psychological constructs underlying health-related attitudes. Divergent validity analyses further supported the conceptual specificity of the questionnaire, as evidenced by the absence of strong and systematic associations with empathy-related dimensions, indicating that the instrument captures constructs that are distinct from general interpersonal sensitivity. To address potential measurement biases, DIF analyses were performed across gender, age, educational level, and administration format. Although DIF emerged as a function of age for 13 items, of educational level for 4 items, and of administration format for 3 items, item functioning was largely comparable for most part of the questionnaire. Indeed, the presence of item-level DIF, particularly in relation to age, suggests that the observed group differences in some subscale scores should be interpreted cautiously, as they may partly reflect differences in item functioning rather than true differences in the underlying constructs. Nevertheless, these results also suggest that observed differences across completion modes are not driven by widespread structural measurement artifacts, but rather by minor, item-specific variations. Consequently, the PF-HBQ and its subscales can be considered stable and comparable across diverse demographic profiles and administration conditions, supporting their broad applicability in both research and healthcare settings.

An important aspect emerging from the present findings concerns the relationship between psychological factors, vaccine hesitancy, and actual vaccination behavior. Results from Study 2 showed that the psychological dimensions assessed by the present questionnaire were able to meaningfully distinguish between individuals with higher versus lower levels of vaccine hesitancy, as defined by a validated measure of vaccine attitudes (VAX-I). Specifically, individuals scoring higher on the VAX-I reported higher levels of Suspiciousness, Extraversion, and Obsessive-Compulsive Traits, along with lower Emotional Stability as measured by the newly developed *PF-HBQ*. However, when individuals were categorized based on vaccination behavior (VAX vs. UNVAX), the same psychological factors were not able to discriminate groups. This pattern suggests that the identified psychological factors are associated with negative attitudes toward vaccination, rather than with vaccination behavior per se. In this sense, the present questionnaire appears to tap into psychological configurations that are aligned with attitudinal hesitancy as conceptualized in the existing literature. Importantly, this lack of differentiation was not limited to the present instrument but was also observed for the VAX-I scores, which although capturing important attitudinal dimensions, such as mistrust of vaccine benefits, concerns about side effects, and preference for natural immunity, did not differentiate vaccinated and unvaccinated individuals. Taken together, these findings suggest that both the newly developed *PF-HBQ* and established measures of vaccine hesitancy capture attitudinal dispositions that do not necessarily translate directly into observable behavior, as the relationship between hesitancy and vaccination uptake is complex and may vary depending on contextual and population-specific factors (64).

This dissociation highlights a critical distinction between vaccine hesitancy as a psychological construct and vaccination status as a behavioral outcome. While hesitancy reflects underlying cognitive–affective and dispositional tendencies, vaccination behavior is likely influenced by a broader set of contextual and structural factors, including access to healthcare, institutional requirements, social norms, and situational constraints (15, 65). For instance, individuals may hold skeptical or ambivalent attitudes toward vaccination while still adhering to vaccination programs due to external pressures or practical considerations. Conversely, some individuals with relatively low hesitancy may remain unvaccinated due to logistical barriers or contextual limitations.

In light of this distinction, future research should further investigate the mechanisms linking psychological vulnerability to vaccination behavior, for instance by incorporating contextual variables, longitudinal designs, and more fine-grained measures of vaccine-related decision processes. Such approaches may help clarify under which conditions psychological factors translate into actual vaccine refusal or acceptance, thereby improving the predictive validity and practical utility of psychological assessments in this domain. Beyond the specific context of vaccination, the present findings also suggest the broader potential applicability of the PF-HBQ in healthcare settings. The questionnaire may represent a useful tool for exploring psychological profiles that influence how individuals engage with medical information, perceive health-related risks, and approach decisions regarding preventive and therapeutic interventions. In this sense, the PF-HBQ could be considered for use across different medical domains to examine individual differences in healthcare engagement, treatment adherence, and responses to clinical recommendations (9). By capturing vulnerability-related patterns and dispositional tendencies, the PF-HBQ may offer a complementary perspective for understanding patient behavior and could potentially support more effective communication strategies and intervention planning.

### 6.1 Limitations

Several limitations of the study should be considered. First, although both exploratory and confirmatory analyses supported the factorial structure, some dimensions were defined by a relatively limited number of items. While this satisfies minimum psychometric requirements, future research might benefit from expanding item pools to more comprehensively capture complex psychological constructs. Second, the factors identified in the final model showed moderate intercorrelations. Although this pattern may reflect the inherently interconnected nature of psychological determinants of health-related attitudes, it may also indicate partial conceptual overlap between some latent dimensions. Third, despite the broad initial item pool, some psychological constructs identified as relevant in previous literature, such as specific dimensions of personality or narcissistic traits (4, 9), were not retained in the final model. This suggests that further refinement of the instrument may be necessary to fully represent the range of psychological factors associated with medical decision-making. Furthermore, the results of Study 2 highlighted a limitation concerning the relationship between psychological factors and vaccination behavior. While the identified dimensions were associated with vaccine hesitancy as measured by a validated attitudinal scale (VAX-I), no differences emerged when considering vaccination status. This may reflect the complexity of the relationship between psychological predispositions and behavior but also suggests that dichotomous indicators of vaccination status may not be sufficiently sensitive to capture variability in vaccine-related decision-making. Additionally, these results should be cautiously interpreted due to unbalanced group sizes (VAX vs. UNVAX). Indeed, logistic regression and cross-sectional analyses, do not allow for causal interpretations regarding the directionality of the observed association. Furthermore, in the present study the UNVAX group was heterogeneous as it included participants who had received only some, or none, of the recommended vaccinations, and in some cases had not received all the mandatory vaccinations. As a result, the use of a dichotomous variable may have oversimplified meaningful differences within this group. Future longitudinal designs are needed to clarify whether specific psychological profiles prospectively predict changes in vaccine hesitancy and related behaviors over time.

## 7 Conclusion

In conclusion, the present study provides evidence supporting the reliability and validity of a novel Italian questionnaire assessing psychological factors associated with health-related decision-making. The *PF-HBQ* allows for the investigation of a range of cognitive, emotional, and dispositional variables that may influence how individuals engage with medical information, perceive health-related risks, and approach preventive and therapeutic interventions. Thus, the *PF-HBQ* provides a useful contribution to the assessment of psychological factors underlying healthcare behavior, complementing existing attitudinal measures.

In the present work, the application of the questionnaire specifically focused on vaccine hesitancy as a relevant and well-established healthcare context. Within this domain, the findings highlight how broader psychological configurations, including affective vulnerability, mistrust-related cognitive tendencies, and dispositional traits, are associated with individual differences in vaccine-related attitudes. Conceptualizing vaccine hesitancy as a psychologically embedded phenomenon may facilitate the identification of vulnerability profiles that shape how individuals interpret health information and respond to preventive interventions. In this perspective, integrating psychologically informed approaches into public health strategies may enhance the effectiveness of interventions aimed at reducing vaccine hesitancy, complementing traditional informational campaigns and addressing the broader emotional and cognitive contexts in which vaccination decisions are made (66).

## Data Availability

The raw data supporting the conclusions of this article will be made available by the authors, without undue reservation.
