# Supplementary material for: Assessing the psychological factors associated with vaccine hesitancy: psychometric properties of the psychological factors in healthcare behavior questionnaire (PF-HBQ)
Source: Front Public Health. 2026 Sep 18;14:1864623. doi: 10.3389/fpubh.2026.1864623 (PMC13630631; doi:10.3389/fpubh.2026.1864623)
Supplement: Supplementary file 1 [file Data_Sheet_1.pdf]

Supplementary Materials

Initial interview for demographic characteristics and medical conditions

Indicate your age: _____ years old

Indicate your gender:

- ☐ Male
- ☐ Female
- ☐ Prefer not to answer

Indicate your educational level:

- ☐ Elementary
- ☐ Middle School
- ☐ High School
- ☐ Bachelor's Degree
- ☐ Master's Degree
- ☐ PhD or Medical Specialization

If you have obtained at least a high school diploma, please indicate in which field (e.g., scientific diploma, degree in nursing, etc.):

____

Do you belong to a religious group?

- ☐ Yes
- ☐ No

If yes, which one?

____

What is your economic status?

- ☐ Less than €20.000
- ☐ €20.000 - €40.000
- ☐ €40.000 - €60.000
- ☐ More than €60.000

What is your marital status?

- ☐ Single
- ☐ Married or in a civil union
- ☐ Widowed
- ☐ Divorced

What is your occupation?

____

Do you live in a city or in a rural area?

- ☐ In a large urban center
- ☐ In a small urban center
- ☐ In a rural area

Have you received the mandatory vaccinations?

- ☐ Yes
- ☐ Some
- ☐ No

If you answered “some” or “none”, why?

____

____

Have you received all the recommended vaccinations for your conditions? (e.g. Covid, influenza, HPV or herpes zooster)

- ☐ Yes
- ☐ No

Do you belong to a high-risk group? (e.g. immunodeficiency or drug allergies)

- ☐ Yes
- ☐ Yes and someone in my household is also high-risk
- ☐ No
- ☐ No, but someone in my household is high-risk

Do you have any medical conditions (e.g. hypertension, diabetes or hypercholesterolemia)?

- ☐ Yes
- ☐ No

If yes, which one?

____

Have you ever experienced adverse reactions to vaccines (serious reactions or mild symptoms)?

- ☐ Yes
- ☐ No

Initial interview for demographic characteristics and medical conditions – Original Italian Version

Indichi la sua età anagrafica: _____ anni

Indichi il suo genere:

- ☐ Maschio
- ☐ Femmina
- ☐ Preferisco non rispondere

Indichi il suo titolo di studio:

- ☐ Licenza elementare
- ☐ Licenza media
- ☐ Diploma di scuola superiore
- ☐ Laurea triennale
- ☐ Laurea magistrale
- ☐ Dottorato di ricerca o titolo specializzazione medica

Se ha conseguito almeno il diploma, indichi in quale ambito (es. diploma scientifico, laurea in scienze infermieristiche ecc.):

____

Appartiene ad un gruppo religioso?

- ☐ Sì
- ☐ No

Se sì, quale?

____

Qual è il suo status economico?

- ☐ Meno di €20.000
- ☐ €20.000 - €40.000
- ☐ €40.000 - €60.000
- ☐ Oltre €60.000

Qual è il suo stato civile?

- ☐ Nubile/Celibe
- ☐ Sposato/a o in unione civile
- ☐ Vedovo/a
- ☐ Divorziato/a

Qual è la sua occupazione?

____

Abita in città o in aree rurali?

- ☐ In un grande centro urbano
- ☐ In un piccolo centro urbano
- ☐ In un'area rurale

Ha fatto i vaccini obbligatori?

- ☐ Sì
- ☐ Alcuni
- ☐ No

Se ha risposto alcuni, oppure nessuno, perché?

____

____

Ha fatto vaccini consigliati per la sua condizione? (es. Covid, influenza, HPV, herpes zooster)

- ☐ Sì
- ☐ No

Fa parte di una categoria di soggetti a rischio? (es. immunodeficienza, allergie a farmaci)

- ☐ Sì
- ☐ Sì e anche qualcuno del mio nucleo familiare è un soggetto a rischio
- ☐ No
- ☐ No, ma qualcuno del mio nucleo familiare è un soggetto a rischio

Presenta condizioni mediche particolari (es. ipertensione, diabete, ipercolesterolemia)?

- ☐ Sì
- ☐ No

Se sì, quali?

____

Ha mai avuto reazioni avverse ai vaccini (reazioni gravi o sintomi lievi)?

- ☐ Sì
- ☐ No

-------- Psychological Factors in Healthcare Behaviour Questionnaire (PF-HBQ) ---------

Istruzioni

Di seguito troverai una serie di affermazioni relative a pensieri, emozioni e atteggiamenti che le persone possono sperimentare nella vita quotidiana. Ti chiediamo di indicare quanto sei d'accordo (da "Completamente in disaccordo" a "Completamente d'accordo") con ciascuna affermazione, selezionando la risposta che meglio descrive il tuo modo di pensare o di sentirti. Non esistono risposte giuste o sbagliate. È importante rispondere in modo spontaneo e sincero, scegliendo l'opzione che rappresenta meglio la tua opinione o esperienza personale.
La compilazione del questionario richiede generalmente circa 15-20 minuti.

Instructions

Below you will find a series of statements related to thoughts, emotions, and attitudes that people may experience in everyday life. Please indicate the extent to which you agree (from "Strongly disagree" to "Strongly agree") with each statement by selecting the response that best reflects how you think or feel. There are no right or wrong answers. It is important to respond spontaneously and honestly, choosing the option that best represents your personal opinion or experience.
Completing the questionnaire generally takes approximately 15–20 minutes.

Item	Original Italian version	English translation
**1**	Sono una persona piuttosto sbadata	I am a rather careless person
**2**	Non mi interessa fare nuove esperienze e conoscere nuove persone	I am not interested in having new experiences or meeting new people
**3**	Penso che si stia complottando contro di me	I think there is a conspiracy against me
**4**	Sono una persona fredda e distaccata	I am a cold and detached person
**5**	Mi è capitato di sentirmi perseguitato/a	I have felt persecuted
**6**	Non mi piace stare in mezzo a molte persone	I do not like being around many people
**7**	Non riesco a scacciare pensieri, parole o idee indesiderate	I cannot get rid of unwanted thoughts, words, or ideas
**8**	Sono convinto/a che gli altri non apprezzino il mio lavoro	I am convinced that others do not appreciate my work
**9**	Sono una persona timida e introversa	I am a shy and introverted person
**10**	Faccio fatica a sentirmi rilassato/a	I find it difficult to feel relaxed
**11**	Ho una forte e vivida immaginazione	I have a strong and vivid imagination
**12**	A volte sento che nulla mi appassiona	Sometimes I feel that nothing interests me
**13**	Mi piace dedicare tempo ai miei hobby	I like to spend time on my hobbies

**14**	Anche se non ho molte cose da fare, ho la sensazione di non poter star dietro a tutto	Even when I do not have much to do, I feel like I cannot keep up with everything
**15**	Sento di non avere le risorse per gestire i miei problemi	I feel that I do not have the resources to manage my problems
**16**	Quando esco di casa controllo finestre, rubinetti del gas e interruttori della luce	When I leave home, I check windows, gas taps, and light switches
**17**	Sono triste per la maggior parte del tempo	I feel sad most of the time
**18**	Ho l'impressione che gli altri possano approfittarsi di me	I have the impression that others may take advantage of me
**19**	Ho spesso voglia di piangere	I often feel like crying
**20**	Sono un vulcano di idee	I am full of ideas
**21**	Tendo a essere una persona riservata	I tend to be a reserved person
**22**	Sono una persona tendenzialmente estroversa	I tend to be an extroverted person
**23**	Tendo a rimandare i miei impegni	I tend to postpone my commitments
**24**	Mi stanco molto mentalmente	I get mentally tired very easily
**25**	Gestisco bene le mie emozioni	I manage my emotions well
**26**	Sono un/a chicchierone/a	I am talkative
**27**	Quando inizio un compito, generalmente lo porto a termine	When I start a task, I usually complete it
**28**	Mi capita di ripetere mentalmente parole, numeri o frasi che interferiscono con l'attività che sto svolgendo	I find myself mentally repeating words, numbers, or phrases that interfere with what I am doing
**29**	Ho la sensazione che le persone mi guardino e parlino male di me	I feel that people look at me and speak badly about me
**30**	Penso che il mio futuro sia privo di speranza	I think my future is hopeless
**31**	Sono stato/a minacciato/a con un'arma	I have been threatened with a weapon
**32**	Sono stato/a aggredito/a fisicamente	I have been physically assaulted
**33**	Non ho fiducia negli altri e nelle loro intenzioni	I do not trust others or their intentions
**34**	Se penso ai miei problemi mi sento molto teso	When I think about my problems, I feel very tense
**35**	Sono pigro/a	I am lazy
**36**	Penso che gli altri vogliano farmi del male	I think others want to harm me
**37**	Sono una persona poco organizzata	I am a disorganized person
**38**	Ho il bisogno di ripetere lo stesso atto come toccare, contare e/o lavarmi le mani	I feel the need to repeat the same actions such as touching, counting, and/or washing my hands
**39**	Ho temuto di avere un attacco di panico	I have feared having a panic attack
**40**	Sono stressato/a	I am stressed
**41**	Sono una persona tranquilla	I am a calm person
**42**	Riesco a rimanere calmo/a anche in situazioni difficili	I am able to remain calm even in difficult situations
**43**	Sono stato/a coinvolto/a in un incidente	I have been involved in an accident
**44**	La mia vita non ha significato	My life has no meaning

**45**	Mi capita di pensare ad un evento negativo di cui sono stato partecipe	I find myself thinking about a negative event I have experienced
**46**	In situazioni sociali, tendo a parlare poco	In social situations, I tend to speak little
**47**	Mi distraggo facilmente	I get distracted easily
**48**	Sono molto fantasioso/a e ingegnoso/a	I am very imaginative and resourceful
**49**	Sono stato/a coinvolto/a in un evento in cui la mia vita è stata in pericolo	I have been involved in an event where my life was in danger
**50**	Sono sicuro/a di non potermi fidare degli altri	I am sure that I cannot trust others
**51**	Per me è difficile fidarmi degli altri	It is difficult for me to trust others
**52**	Mi definisco una persona con una grande inventiva	I consider myself a very inventive person
**53**	Potrei essere un/a bravo/a leader	I could be a good leader

Istruzioni per lo scoring

Assegnare un punteggio grezzo a ciascuna voce secondo la seguente scala: “Completamente in disaccordo = 1”, “Abbastanza in disaccordo = 2”, “Né d’accordo né in disaccordo = 3”, “Abbastanza d’accordo = 4”, “Completamente d’accordo = 5”.
Calcolare i punteggi invertiti per i seguenti item utilizzando la formula [6 − punteggio grezzo]: Item 2, Item 4, Item 6, Item 9, Item 21 e Item 27.

Calcolare la media delle voci per ciascun fattore. Punteggi più elevati indicano livelli più elevati del corrispondente tratto psicologico o di personalità.

1. **Sospettosità (Suspiciousness, S)** – ideazione paranoide, sospettosità, sfiducia
   Item 3 + Item 5 + Item 8 + Item 18 + Item 29 + Item 33 + Item 36 + Item 50 + Item 51 / 9 =
2. **Disagio Generale (General Distress, GD)** – disagio psicologico, vulnerabilità allo stress e sintomi emotivi negativi
   Item 7 + Item 10 + Item 19 + Item 24 + Item 34 + Item 39 + Item 40 + Item 45 / 8 =
3. **Estroversione (Extraversion, E)** – propensione alla socievolezza, all’affettività positiva e all’interazione con gli altri
   Item 2 + Item 4 + Item 6 + Item 9 + Item 21 + Item 22 + Item 26 + Item 46 / 8 =
4. **Apertura (Openness, O)** – propensione alla curiosità, apertura alle esperienze e flessibilità cognitiva
   Item 11 + Item 13 + Item 20 + Item 48 + Item 52 + Item 53 / 6 =
5. **Procrastinazione (Procrastination, P)** – tendenza a rimandare i compiti, difficoltà nell’avviare o portare a termine le attività ed evitamento delle responsabilità
   Item 1 + Item 14 + Item 23 + Item 27 + Item 35 + Item 37 + Item 47 / 7 =
6. **Sintomi depressivi (Depressive Symptoms, DS)** – tendenza all’umore depressivo, alla ridotta motivazione e all’affettività negativa
   Item 12 + Item 15 + Item 17 + Item 30 + Item 44 / 5 =

7. **Sintomi post-traumatici (Post-Traumatic Symptoms, PTS)** – presenza di esperienze psicologiche legate a sintomi traumatici o post-traumatici
   Item 31 + Item 32 + Item 43 + Item 49 / 4 =
8. **Tratti ossessivo-compulsivi (Obsessive-Compulsive Traits, OCT)** – presenza di pensieri ossessivi e tendenze compulsive
   Item 16 + Item 28 + Item 38 / 3 =
9. **Stabilità emotiva (Emotional Stability, ES)** – tendenza all'equilibrio emotivo, sopportazione dello stress ed efficace regolazione emotiva
   Item 25 + Item 41 + Item 42 / 3 =

Scoring Instructions

Assign raw scores to each item according to the following scale: "Strongly disagree = 1", "Somewhat disagree = 2", "Neither agree nor disagree = 3", "Somewhat agree = 4", "Strongly agree = 5".
Compute reverse scores for the following items using the formula [6 − raw score]: Item 2, Item 4, Item 6, Item 9, Item 21 and Item 27.
Calculate the mean of the items for each factor. Higher scores indicate higher levels of the corresponding psychological or personality trait.

1. **Suspiciousness (S)** – paranoid ideation, suspiciousness, mistrust
   Item 3 + Item 5 + Item 8 + Item 18 + Item 29 + Item 33 + Item 36 + Item 50 + Item 51 / 9 =
2. **General Distress (GD)** – psychological distress, vulnerability to stress and negative emotional symptoms
   Item 7 + Item 10 + Item 19 + Item 24 + Item 34 + Item 39 + Item 40 + Item 45 / 8 =
3. **Extraversion (E)** – tendency toward sociability, positive affect, and engagement with others
   Item 2 + Item 4 + Item 6 + Item 9 + Item 21 + Item 22 + Item 26 + Item 46 / 8 =
4. **Openness (O)** – tendency toward curiosity, openness to experiences, and cognitive flexibility
   Item 11 + Item 13 + Item 20 + Item 48 + Item 52 + Item 53 / 6 =
5. **Procrastination (P)** – tendency toward delaying tasks, difficulty initiating or completing activities, and avoidance of responsibilities
   Item 1 + Item 14 + Item 23 + Item 27 + Item 35 + Item 37 + Item 47 / 7 =
6. **Depressive Symptoms (DS)** – tendency toward depressive mood, reduced motivation, and negative affect
   Item 12 + Item 15 + Item 17 + Item 30 + Item 44 / 5 =
7. **Post-Traumatic Symptoms (PTS)** – presence of psychological experiences related to traumatic or post-traumatic symptoms
   Item 31 + Item 32 + Item 43 + Item 49 / 4 =

8. **Obsessive-Compulsive Traits (OCT)** – presence of obsessive thoughts and compulsive tendencies
   Item 16 + Item 28 + Item 38 / 3 =
9. **Emotional Stability (ES) –** tendency toward emotional balance, resilience to stress, and effective emotional regulation
   Item 25 + Item 41 + Item 42 / 3 =

.

Table S1. Differential item functioning by sex

Subscale	Item	Omnibus χ^2	Omnibus pBH	Uniform χ^2	Uniform pBH	Non-uniform χ^2	Non-uniform pBH	Classification
**Suspiciousness**	Item6	3.805	.401					No DIF
	Item8	.178	.915					No DIF
	Item18	2.860	.431					No DIF
	Item44	.682	.800					No DIF
	Item73	.724	.800					No DIF
	Item80	6.755	.307					No DIF
	Item84	1.731	.631					No DIF
	Item115	3.451	.401					No DIF
	Item117	4.475	.401					No DIF
**General Distress**	Item15	.959	.707					No DIF
	Item25	8.770	.050					No DIF
	**Item47**	**23.589**	**< .001**	**18.341**	**< .001**	**5.249**	**.088**	**Uniform DIF**
	Item58	5.570	.099					No DIF
	Item81	2.122	.462					No DIF
	Item92	5.849	.099					No DIF
	Item94	.392	.822					No DIF
	Item106	6.578	.099					No DIF
**Extraversion**	Item5	2.793	.495					No DIF
	**Item7**	**13.046**	**.012**	**12.817**	**.003**	**.228**	**.723**	**Uniform DIF**
	Item14	1.127	.650					No DIF
	Item22	.558	.757					No DIF
	**Item50**	**8.929**	**.031**	**.591**	**.590**	**8.338**	**.031**	**Non-uniform DIF**
	Item51	2.055	.573					No DIF
	**Item69**	**9.014**	**.031**	**4.582**	**.129**	**4.432**	**.141**	**Overall DIF**
	Item107	1.629	.590					No DIF
**Openness**	Item27	.179	.914					No DIF
	Item30	2.381	.914					No DIF
	Item49	.404	.914					No DIF
	Item111	.261	.914					No DIF
	Item119	.900	.914					No DIF
	Item121	.551	.914					No DIF

**Procrastination**	Item3	5.577	.144					No DIF
	Item31	1.723	.493					No DIF
	Item53	4.525	.146					No DIF
	Item70	4.666	.146					No DIF
	Item83	6.118	.144					No DIF
	Item85	5.848	.144					No DIF
	Item109	.661	.719					No DIF
**Depressive Symptoms**	Item29	2.090	.453					No DIF
	Item32	8.480	.072					No DIF
	Item40	1.370	.503					No DIF
	Item74	2.030	.453					No DIF
	Item105	4.730	.235					No DIF
**Post-Traumatic Symptoms**	Item77	2.930	.232					No DIF
	**Item78**	**11.150**	**.008**	**10.362**	**.005**	**.790**	**.499**	**Uniform DIF**
	**Item103**	**16.800**	**< .001**	**5.104**	**.048**	**11.695**	**.003**	**Uniform and non-uniform DIF**
	Item113	3.990	.181					No DIF
**Obsessive-Compulsive Traits**	Item36	3.880	.215					No DIF
	Item72	6.230	.133					No DIF
	Item91	3.040	.218					No DIF
**Emotional Stability**	Item97	.201	.905					No DIF
	Item99	1.428	.905					No DIF
	Item59	.994	.905					No DIF

Note. Two participants who preferred not to report gender were excluded. Cumulative ordinal logistic models were used with the standardized subscale score as the matching criterion. BH-adjusted p values were calculated separately within each subscale. Classification was based on the omnibus BH-adjusted p value; uniform and non-uniform tests were used to characterize omnibus-significant items. Significant DIF were reported in bold.

Table S2. Differential item functioning by age group

Subscale	Item	Omnibus χ^2	Omnibus pBH	Uniform χ^2	Uniform pBH	Non-uniform χ^2	Non-uniform pBH	Classification
**Suspiciousness**	Item6	3.891	.338					No DIF
	Item8	3.751	.338					No DIF
	Item18	.542	.791					No DIF
	Item44	.469	.791					No DIF
	**Item73**	**24.781**	**< .001**	**24.780**	**< .001**	**7.33e-4**	**.978**	**Uniform DIF**
	Item80	1.897	.498					No DIF
	Item84	2.687	.391					No DIF
	**Item115**	**13.165**	**.006**	**12.772**	**.002**	**.393**	**.796**	**Uniform DIF**
	Item117	3.347	.338					No DIF
**General Distress**	Item15	2.462	.467					No DIF
	Item25	2.861	.467					No DIF
	Item47	8.659	.105					No DIF
	Item58	.628	.774					No DIF
	Item81	3.968	.401					No DIF
	Item92	3.790	.401					No DIF
	Item94	1.466	.641					No DIF
	Item106	.512	.774					No DIF
**Extraversion**	**Item5**	**21.220**	**< .001**	**20.315**	**< .001**	**.905**	**.455**	**Uniform DIF**
	**Item7**	**29.591**	**< .001**	**15.475**	**< .001**	**14.116**	**.001**	**Uniform and non-uniform DIF**
	Item14	.971	.615					No DIF
	Item22	5.558	.099					No DIF
	Item50	6.839	.065					No DIF
	**Item51**	**14.439**	**.002**	**3.744**	**.106**	**10.695**	**.004**	**Non-uniform DIF**
	Item69	2.191	.382					No DIF
	Item107	4.725	.126					No DIF
**Openness**	**Item27**	**6.582**	**.031**	**1.113**	**.583**	**6.582**	**.031**	**Non-uniform DIF**
	Item30	1.215	.541					No DIF
	Item49	.249	.741					No DIF
	Item111	.384	.741					No DIF
	Item119	.003	.957					No DIF
	**Item121**	**11.612**	**.004**	**.004**	**.951**	**11.612**	**.004**	**Non-uniform DIF**
**Procrastination**	Item3	.642	.725					No DIF

	Item31	.659	.725					No DIF
	Item53	2.380	.532					No DIF
	Item70	2.805	.532					No DIF
	Item83	.696	.725					No DIF
	**Item85**	**12.860**	**.011**	**8.967**	**.010**	**3.892**	**.340**	**Uniform DIF**
	**Item109**	**10.488**	**.018**	**10.486**	**.008**	**.002**	**.963**	**Uniform DIF**
**Depressive Symptoms**	Item29	.749	.830					No DIF
	Item32	5.160	.189					No DIF
	Item40	2.194	.556					No DIF
	Item74	.374	.830					No DIF
	Item105	7.871	.098					No DIF
**Post-Traumatic Symptoms**	Item77	.236	.889					No DIF
	Item78	4.650	.196					No DIF
	Item103	5.849	.196					No DIF
	Item113	.942	.832					No DIF
**Obsessive-Compulsive Traits**	**Item36**	**23.289**	**< .001**	**15.056**	**< .001**	**8.233**	**.012**	**Uniform and non-uniform DIF**
	**Item72**	**10.493**	**.008**	**10.488**	**.002**	**.004**	**.947**	**Uniform DIF**
	Item91	.649	.723					No DIF
**Emotional Stability**	Item97	1.320	.518					No DIF
	**Item99**	**10.450**	**.008**	**10.438**	**.002**	**.010**	**.919**	**Uniform DIF**
	**Item59**	**18.350**	**< .001**	**17.779**	**< .001**	**.574**	**.673**	**Uniform DIF**

Note. Cumulative ordinal logistic models were used with the standardized subscale score as the matching criterion. BH-adjusted p values were calculated separately within each subscale. Classification was based on the omnibus BH-adjusted p value; uniform and non-uniform tests were used to characterize omnibus-significant items. Significant DIF were reported in bold.

Table S3. Differential item functioning by educational level

Subscale	Item	Omnibus χ^2	Omnibus pBH	Uniform χ^2	Uniform pBH	Non-uniform χ^2	Non-uniform pBH	Classification
**Suspiciousness**	Item6	2.890	.707					No DIF
	Item8	.569	.935					No DIF
	Item18	.371	.935					No DIF
	Item44	.432	.935					No DIF
	Item73	.761	.935					No DIF
	Item80	5.342	.311					No DIF
	Item84	5.837	.311					No DIF
	Item115	2.048	.808					No DIF
	Item117	.130	.937					No DIF
**General Distress**	Item15	.215	.991					No DIF
	Item25	.362	.991					No DIF
	Item47	2.978	.975					No DIF
	Item58	.605	.991					No DIF
	Item81	2.076	.975					No DIF
	Item92	2.011	.975					No DIF
	Item94	.540	.991					No DIF
	Item106	.018	.991					No DIF
**Extraversion**	Item5	4.174	.331					No DIF
	Item7	.490	.783					No DIF
	Item14	2.178	.579					No DIF
	Item22	2.033	.579					No DIF
	Item50	5.997	.199					No DIF
	Item51	9.547	.068					No DIF
	Item69	1.573	.607					No DIF
	Item107	.940	.714					No DIF
**Openness**	Item27	.014	.993					No DIF
	Item30	3.696	.315					No DIF
	Item49	.357	.993					No DIF
	Item111	7.301	.156					No DIF
	Item119	4.771	.276					No DIF
	Item121	2.385	.455					No DIF
**Procrastination**	Item3	.190	.909					No DIF

	**Item31**	**11.055**	**.017**	**10.243**	**.010**	**.812**	**.557**	**Uniform DIF**
	Item53	7.663	.051					No DIF
	Item70	5.137	.134					No DIF
	**Item83**	**10.670**	**.017**	**3.546**	**.209**	**7.124**	**.027**	**Non-uniform DIF**
	Item85	1.008	.846					No DIF
	Item109	.229	.909					No DIF
**Depressive Symptoms**	Item29	5.931	.148					No DIF
	Item32	1.487	.594					No DIF
	Item40	.442	.802					No DIF
	Item74	5.653	.148					No DIF
	Item105	4.765	.154					No DIF
**Post-Traumatic Symptoms**	Item77	1.085	.698					No DIF
	Item78	.718	.698					No DIF
	Item103	.982	.698					No DIF
	Item113	2.733	.698					No DIF
**Obsessive-Compulsive Traits**	Item36	3.933	.210					No DIF
	Item72	.193	.908					No DIF
	Item91	5.985	.150					No DIF
**Emotional Stability**	Item97	4.404	.236					No DIF
	Item99	3.696	.236					No DIF
	Item59	.643	.725					No DIF

Note. Cumulative ordinal logistic models were used with the standardized subscale score as the matching criterion. BH-adjusted p values were calculated separately within each subscale. Classification was based on the omnibus BH-adjusted p value; uniform and non-uniform tests were used to characterize omnibus-significant items. Significant DIF were reported in bold.

Table S4. Differential item functioning by administration format

Subscale	Item	Omnibus χ^2	Omnibus pBH	Uniform χ^2	Uniform pBH	Non-uniform χ^2	Non-uniform pBH	Classification
**Suspiciousness**	Item6	.570	.846					No DIF
	Item8	.681	.846					No DIF
	Item18	.937	.846					No DIF
	Item44	9.198	.080					No DIF
	Item73	1.534	.846					No DIF
	Item80	2.383	.846					No DIF
	Item84	.710	.846					No DIF
	Item115	8.063	.080					No DIF
	Item117	.155	.926					No DIF
**General Distress**	Item15	.473	.790					No DIF
	Item25	1.269	.790					No DIF
	Item47	5.607	.485					No DIF
	Item58	1.347	.790					No DIF
	Item81	.889	.790					No DIF
	Item92	2.305	.790					No DIF
	Item94	.516	.790					No DIF
	Item106	2.720	.790					No DIF
**Extraversion**	Item5	1.223	.664					No DIF
	Item7	3.507	.462					No DIF
	Item14	5.595	.244					No DIF
	Item22	.904	.664					No DIF
	Item50	.819	.664					No DIF
	Item51	6.423	.244					No DIF
	Item69	2.303	.632					No DIF
	Item107	1.133	.664					No DIF
**Openness**	Item27	.094	.954					No DIF
	**Item30**	**10.343**	**.034**	**10.250**	**.008**	**.093**	**.933**	**Uniform DIF**
	Item49	1.054	.886					No DIF
	Item111	6.451	.119					No DIF
	Item119	4.289	.234					No DIF
	Item121	.479	.944					No DIF
**Procrastination**	Item3	1.830	.466					No DIF

	Item31	7.750	.073					No DIF
	**Item53**	**11.500**	**.022**	**.700**	**.564**	**10.804**	**.007**	**Non-uniform DIF**
	Item70	2.370	.466					No DIF
	Item83	6.670	.083					No DIF
	Item85	1.530	.466					No DIF
	Item109	1.730	.466					No DIF
**Depressive Symptoms**	Item29	5.070	.132					No DIF
	Item32	5.220	.132					No DIF
	Item40	1.980	.448					No DIF
	Item74	7.400	.124					No DIF
	Item105	1.610	.448					No DIF
**Post-Traumatic Symptoms**	Item77	1.572	.970					No DIF
	Item78	.256	.983					No DIF
	Item103	.034	.983					No DIF
	Item113	1.447	.970					No DIF
**Obsessive-Compulsive Traits**	Item36	3.130	.449					No DIF
	Item72	.521	.771					No DIF
	Item91	2.413	.449					No DIF
**Emotional Stability**	Item97	3.420	.181					No DIF
	Item99	5.680	.088					No DIF
	**Item59**	**12.160**	**.007**	**12.100**	**.002**	**.054**	**.816**	**Uniform DIF**

Note. Cumulative ordinal logistic models were used with the standardized subscale score as the matching criterion. BH-adjusted p values were calculated separately within each subscale. Classification was based on the omnibus BH-adjusted p value; uniform and non-uniform tests were used to characterize omnibus-significant items. Significant DIF were reported in bold.
